# Factors Associated with Quality of Life in Patients with Dementia with Lewy Bodies: Additional Analysis of a Cross-Sectional Study

**DOI:** 10.3233/JAD-231302

**Published:** 2024-07-16

**Authors:** Shunji Toya, Mamoru Hashimoto, Yuta Manabe, Hajime Yamakage, Manabu Ikeda

**Affiliations:** a Medical Science, Sumitomo Pharma Co., Ltd., Tokyo, Japan; b Department of Psychiatry, Osaka University Graduate School of Medicine, Osaka, Japan; c Department of Neuropsychiatry, Kindai University Faculty of Medicine, Osakasayama, Japan; d Department of Advanced Clinical Medicine, Division of Dementia and Geriatric Medicine, Kanagawa Dental University School of Dentistry, Yokosuka, Japan; e Insight Clinical Development Group, 3H Medi Solution Inc., Tokyo, Japan

**Keywords:** Activities of daily living, Alzheimer’s disease, cross-sectional studies, dementia, depression, hallucinations, Lewy body, parkinsonism, quality of life

## Abstract

**Background:**

Quality of life (QOL) and treatment needs of patients with dementia with Lewy bodies (DLB) and their caregivers are important factors to consider when developing treatment strategies.

**Objective:**

To investigate factors associated with QOL in patients with DLB, and to examine factors associated with activities of daily living (ADL) if ADL was associated with QOL.

**Methods:**

We previously conducted a questionnaire survey study to investigate the treatment needs of patients with DLB and their caregivers. This pre-specified additional analysis evaluated the Physical Component Score (PCS) and Mental Component Score (MCS) of the Short Form-8 for QOL, and the Movement Disorder Society-Unified Parkinson’s Disease Rating Scale (MDS-UPDRS) Part II total score for ADL.

**Results:**

In total, 231 patient– caregiver pairs and 38 physicians were included. Multivariable analysis of QOL showed that the MDS-UPDRS Part II total score (standard regression coefficient [β], – 0.432) was associated with the PCS, and presence of depression (β, – 0.330) was associated with the MCS. The severity of postural instability/gait disorder (PIGD) (β, 0.337) and rigidity (β, 0.266), presence of hallucinations (β, 0.165), male sex (β, 0.157), and use of “short stay” or “small-scale, multifunctional home care” (β, 0.156) were associated with worsened ADL.

**Conclusions:**

In patients with DLB, QOL was negatively impacted by severity of ADL disability and depression, and ADL was negatively impacted by severity of PIGD and rigidity, hallucinations, male sex, and use of “short stay” or “small-scale, multifunctional home care.”

## INTRODUCTION

After Alzheimer’s disease (AD), Lewy body dementia is the second most common form of primary neurodegenerative dementia, which comprises dementia with Lewy bodies (DLB) and Parkinson’s disease dementia [1–3]. DLB, a neurodegenerative form of dementia, is preceded by cognitive impairment with core features including cognitive fluctuations, specific and reproducible visual hallucinations, parkinsonism, rapid eye movement sleep behavior disorder, and sometimes delusions, autonomic dysfunction, sleep disturbances, and depression [4].

In general, quality of life (QOL) is considered to be the quality of an individual’s daily life and has a broad context including emotional, social, and physical aspects [5]. QOL is a key outcome of health and social interventions and is often assessed by evaluating how patients are affected over time by a disease, disability, or disorder. In patients with DLB, QOL is a critical outcome. Currently, clinical evidence regarding the QOL of patients with DLB is limited [6, 7], and there is no formally validated index to assess QOL in patients with DLB. Previous studies have reported that QOL according to the EQ-5D and QOL-AD is lower in patients with DLB than in those with AD [8, 9]. In a mixed population of patients with DLB and AD, depression, anxiety, disability assessment for dementia, and Geriatric Depression Scale scores were associated with QOL [10]. Although there are few relevant published studies in patients with DLB only, Neuropsychiatric Inventory (NPI) scores, independence of instrumental activities of daily living (ADL), caregiver living with the patient, apathy, delusion, depression, constipation, lower instrumental ADL, age, parkinsonism, hallucinations, cognitive fluctuations, and daytime sleepiness are reportedly associated with QOL [8, 9, 11, 12].

In patients with dementia other than DLB, ADL was identified as a key factor related to QOL [13]; thus, improving ADL is necessary to improve QOL in this patient population. ADL can be classified as basic ADL, which are needed to manage basic physical needs such as eating, changing clothes, and bathing, and as instrumental ADL, which are needed to live independently in the community and include activities such as housework, transportation, finance management, and hobbies [14]. Patients with DLB have more impaired function both in basic and instrumental ADL than patients with AD and the same degree of cognitive decline [15, 16]. Furthermore, a previous study conducted in South America found that patients with Lewy body dementia had higher behavioral burden and were less independent in regard to basic and instrumental ADL than patients with AD [17]. However, as with QOL, few studies have examined factors associated with ADL in patients with DLB only. Previous studies have shown that instrumental ADL is associated with Mini-Mental State Examination (MMSE) scores, parkinsonism, hallucinations, cognitive fluctuations, and depressive symptoms in patients with DLB [9, 18].

Previous studies have evaluated QOL and ADL in patients with DLB but have not included information such as communication patterns between attending physicians and patients, or the use of social resources by patients. These factors may also affect QOL and ADL in patients with DLB. Patients rely on and visit their physicians to improve their QOL and ADL, and effective communication between patients and physicians is important for a dementia diagnosis [19]; thus, a positive patient– physician relationship may improve patients’ QOL and ADL. Considering this, there remains a need to clarify the factors associated with QOL and ADL in patients with DLB only.

We have previously investigated and reported on the treatment needs of patients with DLB and their caregivers, as well as their physicians’ understanding of their treatment needs [20]. The attending physicians need to formulate treatment strategies that consider patients’ QOL as well as patients’ and caregivers’ individual treatment needs such as those related to parkinsonism, cognitive impairment, and psychiatric symptoms. In this additional analysis, we investigated a total of 28 factors that could impact QOL in patients with DLB using datasets collected in the main study. We selected the possible factors related to QOL based on previous reports of DLB and other dementias [8–11] and also included patient-physician communication and caregiving (nursing) services based on daily clinical practice. Furthermore, we investigated factors associated with ADL if ADL was identified as a factor associated with QOL.

## MATERIALS AND METHODS

### Study design

Details of the main study design have been reported previously [20]. The main study was a multicenter, cross-sectional, observational, survey study that included patients with DLB, their caregivers, and their attending physicians as the study participants.

This pre-specified additional analysis was conducted in compliance with the ethical principles based on the Declaration of Helsinki (revised in 2013), the Ethical Guidelines for Medical and Health Research Involving Human Subjects (partially revised in 2017), and the research protocol. The study was initially approved by the Ethical Review Board for Observational Research of Osaka University Hospital. In addition, the study was conducted after approval by the respective Ethical Review Committees of each study site. A summary of the main study was registered and published in the UMIN Clinical Trials Registry prior to implementation (UMIN ID: UMIN000041844). Written informed consent was obtained from patients and their caregivers. Physicians consented to participate in the study via the Internet.

### Participants

The inclusion criteria for patients with DLB were being ≥50 years of age with a diagnosis of probable DLB and attending an outpatient clinic. The diagnosis of probable DLB was based on the 2017 consensus report of the DLB Consortium [4]. Patients with Parkinson’s disease with dementia (if parkinsonism had been present for more than 1 year prior to the onset of dementia), patients whose attending physician had not seen them for more than 3 months prior to obtaining consent, and patients who were deemed by the physicians to be unable to complete the questionnaire irrespective of their caregiver’s assistance were excluded. In addition, due to some reason, such as a simple error (forgot to check), there were some patients who were not checked for cognitive impairment by their attending physicians in the cognitive impairment question on the questionnaire. To ensure that patients with DLB were included, such patients who were not checked for cognitive impairment by their attending physician were excluded.

The inclusion criteria for caregivers were being ≥20 years of age and primarily taking care of the patient with DLB. The inclusion criterion for the attending physicians was that they had to be experts in DLB treatment in Japan, defined as previously reported [20].

### Assessments

Questionnaires for patients with DLB and their caregivers were handed out by their attending physician, and patients/caregivers returned the completed questionnaires by mail [20]. The questionnaire for attending physicians was sent by e-mail and answered on the Internet. Before answering the questionnaires, patients were evaluated using the Japanese version of the MMSE (MMSE-J) for cognitive function, the Movement Disorder Society-Unified Parkinson’s Disease Rating Scale (MDS-UPDRS) Part II for ADL, and the MDS-UPDRS Part III for parkinsonism. Caregivers were asked about the behaviors of their patients with DLB using the NPI-12 for behavioral and psychological symptoms of dementia (BPSD) and about cognitive fluctuations in their patients with DLB using the Cognitive Fluctuation Inventory (CFI) [21–24]. The MDS-UPDRS Part II was used to evaluate motor experiences of daily living among the patients with DLB; the questionnaire included the following 13 items: speech; salivation and drooling; chewing and swallowing; eating tasks; dressing; hygiene; handwriting; doing hobbies and other activities; turning in bed; tremor; getting out of bed, car, or deep chair; walking and balance; and freezing.

Patients were also asked to answer the Short Form-8 (SF-8) using a self-completed form for health-related QOL [25]. There is no DLB-specific QOL assessment index; therefore, to assess QOL we used the SF-8, which is a “profile-type scale” that can be easily administered, rather than a “value-type scale” such as the EQ-5D. The SF-8 comprises eight questions covering the following items: general health, physical function, role physical, bodily pain, vitality, social functioning, mental health, and role emotional. In accordance with the manual of the original version of the SF-8 [26], the Physical Component Score (PCS) and the Mental Component Score (MCS) were calculated as indicators of physical and mental QOL, respectively. If a patient was unable to self-complete the SF-8 or had difficulty answering the questionnaires due to parkinsonism or other reasons, his/her caregiver was allowed to fill out the questionnaires by interviewing the patient. In such cases, the caregivers were required to report that they had completed the SF-8 and questionnaire on behalf of the patient.

### Outcomes

The pre-specified outcome measures in this additional analysis study were as follows: 1) for QOL of patients, the PCS and MCS of the SF-8 and 2) for ADL, the MDS-UPDRS Part II total score. Lower PCS and MCS on the SF-8 indicate worse QOL, while higher scores on the MDS-UPDRS Part II total score indicate worse functioning in ADL.

### Statistical analysis

For QOL, the following 28 factors were used as factors influencing the outcome measures: 1) patient’s age (<80, ≥80 years), 2) patient’s sex (female, male), 3) duration after diagnosis of DLB (<24, ≥24 months), 4) duration of education (<12, ≥12 years), 5) patient’s knowledge of DLB (a lot of knowledge, neither yes nor no or does not know very much), 6) frequency of hospital or clinic visits (once every 2– 3 weeks or once every month, once every 2 months or once every 3 months, once every 4 months or more), 7) how well the physician listens to what the patient says (very well or well, normal, not much or not at all or don’t know), 8) patients can talk to anyone other than their physician in hospital or clinic (yes, no or don’t know), 9) number of people living with the patient (alone, ≥2), 10) use of “long-term care” or “outpatient rehabilitation” (no, yes), 11) use of “short stay” or “small-scale, multifunctional home care” (no, yes), 12) MMSE-J total score (<22, ≥22), 13) MDS-UPDRS Part III total score (<18, ≥18), 14) NPI-10 total score (<11, ≥11), 15) nighttime behavior (no; NPI-nighttime behavior score = 0, yes; NPI-nighttime behavior score≥1), 16) appetite (no; NPI-appetite score = 0, yes; NPI-appetite score≥1), 17) cognitive fluctuation (no; CFI score = 0, yes; CFI score≥1), 18) autonomic dysfunction (no, yes), 19) sensory disorder (no, yes), 20) caregiver’s age (<65, ≥65 years), 21) caregiver’s sex (female, male), 22) caregiver’s knowledge of DLB (a lot of knowledge, neither yes nor no or does not know very much), 23) caregiver lives with the patient (no, yes), 24) caregiver’s time spent with the patient (<16, ≥16 hours per day), 25) caregiver’s job status (i.e., concurrently working) (yes, no), 26) caregiver’s relationship with the patient (spouse, non-spouse), 27) person who filled out the SF-8 (patient, caregiver), and 28) MDS-UPDRS Part II total score (<9, ≥9). Factors 1– 26 of the 28 listed above were used to assess patient ADL. The NPI-10 was used by excluding the subitems of “nighttime behavior” and “appetite” from the NPI-12. Continuous variables except NPI-night behavior and appetite score were binarized by the median value. Details of how patients’ and caregivers’ knowledge of DLB (factors 5 and 22), autonomic dysfunction (factor 18), and sensory disorder (factor 19) were evaluated are included in the Supplementary Methods.

Linear regression analysis was used to investigate the factors that had an impact on the outcome measures (patient QOL and ADL). The PCS and MCS from the SF-8 were used as dependent variables to examine factors contributing to the deterioration of QOL, and the MDS-UPDRS Part II total score was used as the dependent variable to examine factors contributing to the deterioration of ADL.

The multivariable model used a stepwise selection method. Specifically, variable selection was performed using the stepwise method after univariable analysis to identify significant factors. Furthermore, when extracting the MDS-UPDRS Part III total score or the NPI-10 total score in each multivariable analysis, we created a new model in which items other than the MDS-UPDRS Part III total score or the NPI-10 that were significant in the univariable analysis, and four symptoms of parkinsonism (tremor [sum of MDS-UPDRS items 3.15–3.18], rigidity [sum of item 3.3], bradykinesia [sum of items 3.4–3.8 and 3.14], and postural instability/gait disorder [PIGD, sum of items 3.9–3.13]) [27] or the NPI-10 subitems that were significant in the univariable analysis were entered by a stepwise method. Rigidity, bradykinesia, and PIGD were binarized by median score of severity, and tremor was binarized by 0 and≥1 because median severity of tremor was 0. Each NPI-subitem was binarized by 0 and≥1. These stepwise multivariable models were selected because this was an exploratory analysis, the number of candidate factors was large relative to the sample size, and the problem of collinearity could be statistically avoided. Factors with statistically significant negative coefficient values in multivariable analyses for PCS and MCS were interpreted as factors associated with worsened physical- and mental-related QOL. Factors with statistically significant positive coefficient values in the multivariable analysis of ADL were interpreted as factors associated with worsened ADLstatus.

Missing data were excluded from the analysis on a missing item basis. In the multivariable analyses, if a variable with missing data was included in the analysis model, it was excluded on a case-by-case basis.

Statistical significance in this study was set at 0.05 (two-sided) unless otherwise noted. When multiple multivariable analyses were performed, the significance level was adjusted for multiplicity by dividing 0.05 by the number of times of multivariable analysis. All analyses were performed using SAS ver. 9.4 (SAS Institute Inc., Cary, NC, USA).

## RESULTS

### Participants’ characteristics

**Fig. 1:**
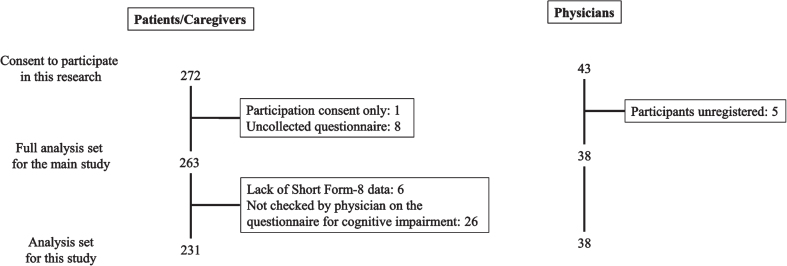
Disposition of the study participants.

This study was conducted at 35 facilities with DLB expert physicians in Japan from September 2020 to July 2021. The disposition of the study participants is shown in Fig. 1. In the main study, a total of 263 pairs of patients with DLB and their caregivers with 38 attending physicians were included in the full analysis set [20]. Of the full analysis set, 231 pairs with 38 attending physicians were included in the analysis set for the present study, excluding six patients who did not provide answers to any of the eight questions of the SF-8, and 26 patients who were not checked on the questionnaire for cognitive impairment by their attending physician.

The background characteristics of the 231 patients and caregivers are shown in Table 1, and those of the 38 attending physicians are shown in Supplementary Table 1. The patients’ age (mean±standard deviation [SD]) was 79.8±6.4 years, the duration after diagnosis of DLB was 30.5±31.1 months, and 45.9% of patients were male. The PCS of the SF-8 as an indicator of physical QOL was 45.9±8.2, and the MCS of the SF-8 as an indicator of mental QOL was 48.8±6.9. The MDS-UPDRS Part II total score as an indicator of ADL was 10.1±10.0. Of the 214 (92.6%) patients living in their own homes, 110 (47.6%) used day care/day services (long-term care or outpatient rehabilitation), and 47 (20.3%) used medical services involving overnight stays (short stays or small-scale, multifunctional home care). The MMSE-J total score was 20.5±5.9, NPI-12 total score was 17.0±17.1, and MDS-UPDRS Part III total score was 22.2±19.8, indicating that patients with mild to moderate DLB were included in this study.

**Table 1:** Background characteristics of patients with DLB and their caregivers (analysis set for factors affecting QOL)

	Total *N* = 231
Patient’s age (y)	79.8±6.4
Patient’s sex (male/female)	106/125
Duration after diagnosis of DLB (months)	30.5±31.1 (*n* = 228)
Education (y)	11.6±2.8 (*n* = 211)
Institute	
University hospital	95 (41.1)
Non-university hospital	50 (21.6)
Clinic	86 (37.2)
Number of people living with the patient in their own home	
Alone	23 (10.0)
Two or more	191 (82.7)
Number of people living in outside their home	17 (7.4)
Use of “long-term care”^a^ or “outpatient rehabilitation”^b^ (yes)	110 (47.6)
Use of “short stay”^c^ or “small-scale, multifunctional home care”^d^ (yes)	47 (20.3)
MMSE-J total score	20.5±5.9
NPI-12 total score	17.0±17.1 (*n* = 229)
MDS-UPDRS Part III total score	22.2±19.8
Tremor	2.5±4.1
Rigidity	3.6±3.8
Bradykinesia	10.0±9.3
PIGD	4.6±4.9
MDS-UPDRS Part II total score	10.1±10.0
CFI	2.3±3.0
Autonomic dysfunction (yes)	104 (45.0)
Sensory disorder (yes)	27 (11.7)
Patient’s SF-8 score	
Physical component score	45.9±8.2
Mental component score	48.8±6.9
Physical functioning	46.2±7.6
Role physical	45.1±10.4
Bodily pain	48.6±10.0
General health	50.8±7.3
Vitality	50.1±6.0
Social functioning	47.0±8.5
Role emotional	48.3±7.8
Mental health	48.6±7.0
Current symptom category^e^	
Cognitive impairment	231 (100.0)
Visual hallucinations	128 (55.4)
Parkinsonism	179 (77.5)
REM sleep behavior disorder	93 (40.3)
Cognitive fluctuation^f^	136 (58.9)
Concomitant drugs	
Cholinesterase inhibitor	201 (87.0)
Memantine	43 (18.6)
Levodopa	73 (31.6)
Zonisamide^g^	16 (6.9)
Dopamine agonist	11 (4.8)
Antipsychotic	42 (18.2)
Yokukansan	44 (19.0)
Antidepressant	30 (13.0)
Anxiolytic^h^	30 (13.0)
Anticonvulsant (not including zonisamide^i^)	8 (3.5)
Clonazepam	19 (8.2)
Hypnotic^j^	66 (28.6)
Anti-orthostatic-hypotension drug^k^	1 (0.4)
Anti-constipation drug^l^	32 (13.9)
Anti-dysuria drug^m^	12 (5.2)
Caregiver’s age (years)	64.9±12.9
Caregiver’s sex (male/female)	64/167
Caregiver lives with the patient (yes)	184 (79.7)
Caregiver’s time spent with the patient (hours/day)	14.3±8.9 (*n* = 227)
Caregiver’s job status (i.e., concurrently working) (yes)	101 (44.1)
Caregiver’s relationship with the patient	
Spouse	116 (50.2)
Non-spouse^n^	115 (49.8)

Data are mean±standard deviation, *n* (%), or *n*. ^a^Refers to a nursing care service in which older individuals who live in their own homes can visit a facility during daytime to receive meals, be bathed, or enjoy recreational activities. ^b^Refers to rehabilitation services (health management services) aimed at maintaining/improving mental and physical functions and supporting self-sustenance in the daily lives of older people. Service users are older people living at home who visit rehabilitation facilities (health facilities for the older, hospitals, clinics, etc.) without staying overnight. ^c^Refers to a nursing care service in which older individuals who live in their own homes can stay in a facility for a few days to receive meals, be bathed, or enjoy recreational activities. ^d^Refers to a service that provides a combination of day services, home care, and short stays to enable people to continue living at home. ^e^Current symptom category based on physician reports (not patient reports). ^f^Patients with CFI score≥1. ^g^Zonisamide is approved for treatment of parkinsonism in patients with DLB in Japan. ^h^Includes lorazepam, clonazepam, clotiazepam, diazepam, etizolam, bromazepam, alprazolam, tandospirone. ^i^Excludes zonisamide and includes valproate sodium and carbamazepine. ^j^Excludes clonazepam and includes ramelteon, zolpidem, zopiclone, eszopiclone, triazolam, etizolam, brotizolam, rilmazafone, lormetazepam, flunitrazepam, estazolam, nitrazepam, quazepam, flurazepam, haloxazolam, suvorexant, and lemborexant. ^k^Includes droxidopa. ^l^Includes linaclotide, elobixibat, magnesium oxide formulation, polyethylene glycol formulation, anthraquinone class, diphenyl class, mosapride, Chinese herbal medicine except irritant laxatives, and other. ^m^Includes imidafenacin, oxybutynin hydrochloride, fesoterodine fumarate, mirabegron, vibegron, and urapidil. ^n^Includes daughters or sons of the patient (*n* = 95, 41.1%), daughters- or sons-in-law (*n* = 12, 5.2%), siblings (*n* = 4, 1.7%), grandchildren (*n* = 2, 0.9%), and care providers (*n* = 2, 0.9%). CFI, Cognitive Fluctuation Inventory; DLB, dementia with Lewy bodies; MDS-UPDRS, Movement Disorder Society-Unified Parkinson’s Disease Rating Scale; MMSE-J, Mini-Mental State Examination, Japanese version; NPI-12, Neuropsychiatric Inventory-12; QOL, quality of life; REM, rapid eye movement; SF-8, Short Form-8.

The caregivers’ age (mean±SD) was 64.9±12.9 years, and most (72.3%) were female. The caregivers lived with the patients in 79.7% of cases, spending approximately 14.3±8.9 hours per day with the patient. The relationship between the caregivers and patients was spouse in 50.2% of cases. Among all 38 physicians, the most common specialty was psychiatry (26 [69.4%]), followed by neurology (6 [15.8%]).

Regarding the SF-8 questionnaire, 53.2% (123/231) of patients filled out the questionnaire themselves and 46.8% (108/231) were assisted in doing so by their caregiver (Supplementary Tables 2 and 3). The mean SF-8 total score was similar between patients who filled out the SF-8 questionnaire by themselves and those who had caregiver assistance (PCS, 46.32 versus 45.33, *p* = 0.356; MCS, 48.98 versus 48.50, *p* = 0.597).

### Factors associated with QOL

#### Factors associated with the PCS of the SF-8

The univariable and multivariable analyses of factors associated with the PCS of the SF-8 are shown in Supplementary Table 2 and Table 2. A multivariable analysis using the stepwise method with the six factors extracted in the univariable analysis showed that the MDS-UPDRS Part II total score was the only extracted factor that was significantly associated with physical-related QOL, with a standard regression coefficient of –0.432 (*p* < 0.001) (Table 2).

**Table 2:** Multivariable analysis of SF-8 PCS (analysis set for factors affecting QOL)

Factors ^a^	B	SE	β	t-statistic	*p* ^b^	VIF
Duration after diagnosis of DLB (mo)	NE					
<24						
≥24						
How well the physician listens to what the patient says	NE					
Very well, well						
Normal						
Not much, not at all, don’t know						
Use of “short stay” or “small-scale, multifunctional home care”	NE					
No						
Yes						
MDS-UPDRS Part III total score	NE					
<18						
≥18						
MDS-UPDRS Part II total score						
<9	ref					
≥9	– 7.017	0.984	– 0.432	– 7.131	<0.001	1.000
NPI-10 total score	NE					
<11						
≥11						

^a^The adjustment factors (independent variables) used in the stepwise method were as follows: duration after diagnosis of DLB, how well the physician listens to what the patient says, use of “short stay” or “small-scale, multifunctional home care,” MDS-UPDRS Part III total score, MDS-UPDRS Part II total score, and NPI-10 total score. ^b^The significance level was < 0.05. B, regression coefficient; MDS-UPDRS, Movement Disorder Society-Unified Parkinson’s Disease Rating Scale; NE, not elected; NPI, Neuropsychiatric Inventory; PIGD, postural instability/gait disorder; PCS, Physical Component Score; ref, reference; QOL, quality of life; SE, standard error; SF-8, Short Form-8; VIF, variance inflation factor; β, standard regression coefficient.

#### Factors associated with the MCS of the SF-8

The univariable analysis with MCS scores as the dependent variable identified three significant variables: frequency of hospital or clinic visits, NPI-10 total score, and cognitive fluctuation (all *p* < 0.05) (Supplementary Table 3). Subsequently, a stepwise multivariable analysis using the three factors extracted from the univariable analysis showed only the NPI-10 total score as a significant factor (*p* < 0.05) (Supplementary Table 4).

As the NPI-10 total score was extracted as a significant variable in the multivariable analysis, a univariable analysis with the 10 subitems of the NPI-10 as the independent variables and with MCS as the dependent variable was performed. Because a total of two multivariable analyses were performed, the statistical significance level was corrected from *p* < 0.05 to *p* < 0.025. Hallucinations and depression were identified as significant variables (both *p* < 0.025) (Supplementary Table 5).

Finally, a multivariable analysis using the stepwise method with four factors (frequency of hospital or clinic visits, hallucinations, depression, and cognitive fluctuation) was performed. Because a total of three multivariable analyses were performed, the statistical significance level was corrected from *p* < 0.05 to *p* < 0.016. Only depression was extracted as a significant factor associated with the MCS, with a standard regression coefficient of –0.330 (*p* < 0.001) (Table 3).

**Table 3:** Multivariable analysis of SF-8 MCS (analysis set for factors affecting QOL)

Factors ^a^	B	SE	β	t-statistic	*p* ^b^	VIF
Frequency of hospital or clinic visits	NE					
Once every 2 to 3 weeks, once every month						
Once every 2 months, once every 3 months						
Once every 4 months or more						
Hallucinations	NE					
No						
Yes						
Depression						
No	ref					
Yes	–4.646	0.895	–0.330	–5.192	<0.001	1.000
Cognitive fluctuation	NE					
No						
Yes						

^a^The adjustment factors (independent variables) used in the stepwise method were as follows: frequency of hospital or clinic visits, hallucinations, depression and cognitive fluctuation. ^b^The significance level was < 0.016. B, regression coefficient; MCS, Mental Component Score; NE, not elected; QOL, quality of life; ref, reference; SE, standard error; SF-8, Short Form-8; VIF, variance inflation factor; β, standard regression coefficient.

### Factors associated with ADL in patients with DLB

As the MDS-UPDRS Part II total score, an indicator of ADL, was extracted as a significant factor associated with physical-related QOL, factors associated with patients’ ADL based on the MDS-UPDRS Part II total score were examined next. The univariable analysis identified 11 significant variables (all *p* < 0.05) (Supplementary Table 6).

Subsequently, a multivariable analysis using the stepwise method with these 11 factors showed that patient’s sex, duration after diagnosis of DLB, use of “short stay” or “small-scale, multifunctional home care,” MDS-UPDRS Part III total score, and NPI-10 total score were significantly associated with ADL (all *p* < 0.05) (Supplementary Table 7).

As the MDS-UPDRS Part III total score and the NPI-10 total score were extracted as significant variables in the multivariable analysis, a univariable analysis with the four symptoms of parkinsonism and the 10 subitems of the NPI-10 as the independent variables was conducted. Based on the adjusted significance level of *p* < 0.025, the severity of tremor, rigidity, bradykinesia, and PIGD, and the presence of hallucinations, agitation, and apathy were extracted as significant variables (Supplementary Table 8).

Finally, multivariable analysis with 16 factors (patient’s age, patients’ sex, duration after diagnosis of DLB, how well the physician listens to what the patient says, use of “long-term care” or “outpatient rehabilitation,” use of “short stay” or “small-scale, multifunctional home care,” MMSE-J total score, severity of tremor, rigidity, bradykinesia, and PIGD, and presence of hallucinations, agitation, apathy, nighttime behavior, and autonomic dysfunction) was performed. The factor associated with ADL that had the highest standard regression coefficient (0.337, *p* < 0.001) was the severity of PIGD. After PIGD, the factors associated with ADL with the second highest regression coefficients were severity of rigidity (0.266, *p* < 0.001), followed by presence of hallucinations (0.165, *p* = 0.001), male sex (0.157, *p* = 0.001), use of “short stay” or “small-scale, multifunctional home care” (0.156, *p* = 0.002), duration after diagnosis of DLB (0.123, *P* = 0.019), and presence of apathy (0.111, *p* = 0.026) (Table 4). Of these, the severity of PIGD, severity of rigidity, presence of hallucinations, male sex, and use of “short stay” or “small-scale, multifunctional home care” reached the adjusted statistical significance level of *p* < 0.016.

**Table 4:** Multivariable analysis of ADL (analysis set for factors affecting ADL)

Factors ^a^	B	SE	β	t-statistic	*p*	VIF
Patient’s age (y)	NE					
<80						
≥80						
Patient’s sex						
Female	ref					
Male	3.109	0.955	0.157	3.256	0.001	1.012
Duration after diagnosis of DLB (mo)						
<24	ref					
≥24	2.417	1.022	0.123	2.365	0.019	1.167
How well the physician listens to what the patient says	NE					
Very well, well						
Normal						
Not much, not at all, don’t know						
Use of “long-term care” or “outpatient rehabilitation”	NE					
No						
Yes						
Use of “short stay” or “small-scale, multifunctional home care”						
No	ref					
Yes	3.768	1.219	0.156	3.091	0.002	1.110
MMSE-J total score	NE					
<22						
≥22						
Tremor	NE					
0						
≥1						
Rigidity						
<3	ref					
≥3	5.247	1.090	0.266	4.813	<0.001	1.327
Bradykinesia	NE					
<9						
≥9						
PIGD						
<3	ref					
≥3	6.721	1.111	0.337	6.050	<0.001	1.347
Hallucinations						
No	ref					
Yes	3.260	0.968	0.165	3.367	0.001	1.047
Agitation	NE					
No						
Yes						
Apathy						
No	ref					
Yes	2.323	1.039	0.111	2.235	0.026	1.070
Nighttime behavior	NE					
No						
Yes						
Autonomic dysfunction	NE					
No						
Yes						

^a^The adjustment factors (independent variables) used in forced entry were as follows in the stepwise method: patient’s age, patient’s sex, duration after diagnosis of DLB, how well the physician listens to what the patient says, use of “long-term care” or “outpatient rehabilitation,” use of “short stay” or “small-scale, multifunctional home care,” MMSE-J total score, severity of tremor, rigidity, bradykinesia and PIGD, hallucinations, agitation, apathy, nighttime behavior, and autonomic dysfunction. ^b^The significance level was < 0.016. ADL, activities of daily living; B, regression coefficient; DLB, dementia with Lewy bodies; MMSE-J, Mini-Mental State Examination; PIGD, postural instability/gait disorder; NE, not elected; ref, reference; SE, standard error; VIF, variance inflation factor; β, standard regression coefficient.

## DISCUSSION

**Fig. 2:**
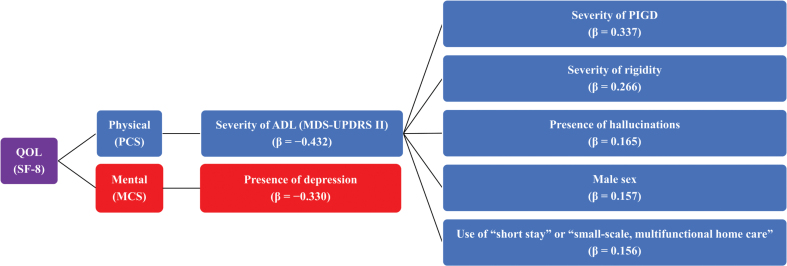
Overview of study results. ADL, activities of daily living; DLB, dementia with Lewy bodies; MCS, Mental Component Score; MDS-UPDRS, Movement Disorder Society-Unified Parkinson’s Disease Rating Scale; PCS, Physical Component Score; PIGD, postural instability/gait disorder; QOL, quality of life; SF-8, Short Form-8; β, standard regression coefficient.

We conducted an additional analysis of a multicenter, cross-sectional, survey study to investigate factors associated with physical- and mental-related QOL and ADL in patients with DLB, such as communication patterns between attending physicians and patients, or factors associated with the use of social resources by patients. A diagram summarizing the overall results of this study is shown in Fig. 2. Briefly, we found that factors associated with the physical-related QOL of patients with DLB were severity of ADL disability and the factor associated with mental-related QOL was presence of depression. We also found that factors associated with ADL in patients with DLB were severity of parkinsonism, such as PIGD and rigidity, presence of hallucinations, male sex, and use of “short stay” and/or “small-scale, multifunctional home care”.

In the present study, the severity of ADL disability was directly associated with the physical component of QOL, and the presence of depression was associated with the mental component of QOL. Our results are generally consistent with previous studies of patients with DLB and other types of dementia, although the indices to measure QOL and ADL used between the present and previous studies were different [8, 10–13]. Boström et al. reported that NPI score, instrumental ADL, delusions, and apathy were associated with QOL in patients with DLB [8]. Another study reported that depression, constipation, and lower instrumental ADL were associated with lower QOL in patients with DLB [11], and ADL has also been identified as a key factor for QOL in patients with dementia [13]. Regarding depression, DLB is associated with a relatively high rate of depression as a complication [28] and patients are at a higher risk of suicide compared with other types of dementia [29]. Furthermore, a previous study reported that older people with depression have decreased walking function and decreased life function [30], which can reduce the activity of patients with DLB and impair their prognosis. In the main study, depression was ranked low in patients’ treatment needs [20]; however, an active therapeutic intervention for depression may also improve QOL in patients with DLB and be necessary regardless of the patient’s own awareness.

The factor most strongly associated with ADL was the severity of PIGD, followed by rigidity; however, other parkinsonism symptoms such as tremor and bradykinesia were not extracted as significant factors after adjustment in the multivariable analysis. In Parkinson’s disease, which is on the same neuropathologic spectrum as DLB, lack of defensive movement (abduction or extension of the upper extremity) is often observed during lateral falls and falls with the upper extremity in adduction or flexion, resulting in direct external force to the femoral condyle and leading to fractures [31]. These severe traumatic injuries reduce patients’ activity level over time [32] and impair cardiopulmonary function, including restricted ventilation and increased susceptibility to aspiration pneumonia, contributing to poor prognoses. Treatment of parkinsonism, especially PIGD and rigidity, can prevent trauma from falls and suppress the worsening of patients’ ADL. Thus, it is considered that treatment of parkinsonism in patients with DLB is important when considering the QOL of patients with DLB. Bradykinesia is more frequent than tremor at rest in patients with DLB [33], and tremor is less frequent in patients with DLB than in patients with Parkinson’s disease [34]. While motor symptoms such as bradykinesia have been associated with ADL and QOL in patients with Parkinson’s disease [35, 36], no such association has been reported in patients with DLB. Further research is needed to understand the severity cut-off scores of the four parkinsonism symptoms (tremor, rigidity, bradykinesia, and postural stability/gait disorder) and to examine the relationship between tremor or bradykinesia and ADL in patients with DLB.

This study also revealed that the presence of hallucinations was associated with impaired ADL. Previous studies reported that patients with DLB had more frequent hallucinations and lower ADL than patients with AD [37] and that hallucinations increased the burden of caregivers of patients with DLB [15]. Although there is little evidence to directly evaluate whether hallucinations are an independent factor associated with impaired ADL in patients with DLB, hallucinations have been previously reported to be associated with impaired ADL in patients with DLB [9], Parkinson’s disease, and AD [38]. It may be assumed that the presence of visual hallucinations is a limiting factor for activities such as hobbies and entertainment. There is currently a lack of scientific evidence on the relationship between these factors in patients with DLB and this should be evaluated in the future. Considering that hallucinations are one of the factors that can disrupt home care, therapeutic intervention is required for those who experience a level of hallucinations that is uncomfortable for the patient and causes difficulties for the caregiver.

Interestingly, the current findings show that male sex was associated with impaired ADL. Utsumi et al. reported that among patients with DLB, the incidence of parkinsonism at the time of diagnosis was higher in men than in women, and hallucinations were more frequent in women than in men [39]. Additionally, Matar et al. reported that the severity of parkinsonism progressed over time, but that the severity of hallucinations was stable over time in patients with DLB [40]. However, in the present study, there were no significant differences in the frequency or severity of parkinsonism or hallucinations between male and female patients (data not shown). Of note, several studies in the general population have reported sex differences in ADL [41, 42]. It is possible that these results of an association between male sex and impaired ADL are not specific to patients with DLB, although the cause of these results is unknown. Alternatively, it is possible that an existing confounding factor in the background characteristics of patients associated with sex, which was not assessed in this study, had an effect on patients’ ADL.

The present study showed that use of “short stay” and/or “small-scale, multifunctional home care” was associated with worsened ADL. We did not examine changes in ADL before and after service use; therefore, our results do not necessarily indicate that the use of “short stay” or “small-scale, multifunctional home care” worsened ADL. Our interpretation is that patients with worsened ADL may tend to use “short stay” or “small-scale, multifunctional home care” services. Longitudinal studies are needed to determine whether the use of these services improves ADL or QOL in patients with DLB.

The results of the present study were consistent with a previous study in that cognitive impairment based on MMSE score was not found to be associated with QOL [9]. In addition, we found no associations between delusions and QOL. One possible reason for this inconsistency is that patients with severe delusions were not included in this study. Another possible reason is that QOL may depend on the patient’s subjective symptoms even when objective assessment tools for QOL are used. Delusions are likely to represent a lack of patients’ awareness of their illness, resulting in an underestimation of the association with QOL.

The present study has some limitations. There is the issue of selection bias. Many patients with DLB who participated in the main questionnaire survey had relatively mild disease, and those who were deemed inappropriate for the survey by their physicians did not participate. Therefore, it is difficult to apply these results to the entire population of patients with DLB. The MDS-UPDRS Part II score was used as the ADL evaluation index and may not have accurately measured instrumental ADL. In this study, the severity of cognitive impairment was measured using the MMSE, which is a useful screening test but may not accurately measure the severity of cognitive impairment in patients with DLB. Although this was a pre-specified additional analysis, this study was not a primary objective in the overall study. We adjusted for multiplicity in the multivariable analyses; however, the univariable analysis did not adjust for multiplicity by repeating the test for the 28 factors. Given the sample size, it was difficult to examine the multiplicity of the 28 factors. Therefore, we did not adjust for multiplicity in the univariable analysis and considered *p* < 0.05 to be a statistically significant difference. We considered whether the use of concomitant medications could be added as a factor for the assessment of caregiver burden, but as this was a cross-sectional study, data on concomitant medications were provided at the time of the questionnaire survey. Therefore, in the multivariable analysis, we were not able to adjust for the presence or absence of concomitant medications. The results of this study are hypothesis-generating and not confirmatory. However, as DLB presents with a variety of symptoms, and each symptom is heterogeneous, it was assumed that various factors are involved in the QOL and ADL of patients, and it was difficult to set a specific hypothesis in advance. The identified factors related to QOL and ADL in patients with DLB in this study should be verified by other assessment tools for QOL and ADL. Our findings should also be verified in a longitudinal study. There is no validated index to measure QOL in patients with DLB, but this study used scores of the eight domains of the SF-8, which is a simple measure of health-related QOL. In this study, several MCS subdomain scores in patients with DLB were lower than the average values for older Japanese people (aged 80 to 85 years), and the PCS and MCS scores in this study were lower than those previously reported for older healthy people (mean age of 76.0±6.9 years) [43]. Furthermore, the QOL-related factors identified in this study were broadly consistent with previous studies, suggesting that the SF-8 score can be used to assess QOL for patients with DLB, although this use has not been validated for patients with DLB. Finally, this study was conducted in the midst of a new COVID-19 outbreak in Japan, which increased the amount of time participants spent at home. Therefore, the QOL and ADL of patients with DLB were likely somewhat affected. Although there are several reports associated with ADL in patients with DLB, reports on QOL are limited. In the future, it will be necessary to investigate the factors associated with QOL and ADL in patients with DLB using a new design that takes these limitations into account.

In conclusion, in patients with DLB, factors that had a negative impact on QOL were the severity of ADL disability and presence of depression; factors that had a negative impact on ADL were severity of PIGD and rigidity, presence of hallucinations, male sex, and use of “short stay” or “small-scale, multifunctional home care.” When considering the QOL of patients with DLB, it is important to focus on evaluating the presence of depression and patients’ ability to perform ADL independently.

## AUTHOR CONTRIBUTIONS

Shunji Toya (Conceptualization; Funding acquisition; Investigation; Methodology; Project administration; Resources; Writing – original draft; Writing – review & editing); Mamoru Hashimoto (Conceptualization; Investigation; Methodology; Project administration; Supervision; Writing – original draft; Writing – review & editing); Yuta Manabe (Conceptualization; Investigation; Methodology; Project administration; Writing – original draft; Writing – review & editing); Hajime Yamakage (Data curation; Formal analysis; Validation; Writing – review & editing); Manabu Ikeda (Conceptualization; Investigation; Methodology; Project administration; Supervision; Writing – original draft; Writing – review & editing).

## Supplementary Material

Supplementary Material

## Data Availability

Owing to participant privacy, individual-level data cannot be made publicly available.
