# Supplementary material for: Factors Associated with Quality of Life in Patients with Dementia with Lewy Bodies: Additional Analysis of a Cross-Sectional Study
Source: J Alzheimers Dis. 2024 Jul 16;100(2):525–38. doi: 10.3233/JAD-231302 (PMC11307033; doi:10.3233/JAD-231302)
Supplement: Supplementary Material [file jad-100-jad231302-s001.pdf]

Supplementary Material

Associations of Plasma Tau with Amyloid and Tau PET: Results from the Community-Based Framingham Heart Study

Supplementary Table 1. Comparison of demographic and clinical characteristics of those enrolled in the PET study versus full Cohort.

**Variable**	**PET Sample (n=425)**	**No-PET sample (n=6008)**	**p**
Age, Mean (SD)	47.6 (9.1)	56.7 (13.6)	**<0.001**
BMI, Mean (SD)	28.1 (5.4)	28.1 (5.7)	0.94
Hypertensive medication, n (%)	79 (19%)	1987 (33%)	**<0.001**
Hypertension, n (%)	105 (25%)	2440 (41%)	**<0.001**
Diabetes, n (%)	21 (5%)	562 (10%)	**0.002**
Male sex, n (%)	212 (50%)	2749 (46%)	0.09
Education, n (%)			
No high-school degree	0 (0%)	152 (3%)	**<0.001**
High-school degree	43 (10%)	1274 (21%)	
Some college	106 (25%)	1771 (30%)	
College graduate	275 (65%)	2749 (46%)	
APOE4, n (%)	95 (23%)	1271 (22%)	0.67

Significant associations highlighted in bold.

Supplementary Table 2. Associations between PET and plasma tau in those with both Aβ and tau PET (n=313)

PET measure (dependent variable)	Plasma tau (independent variable)	
	β (SE)	p
Amyloid FLR	0.07 (0.04)	0.15
Amyloid precuneus	**0.13 (0.05)**	**0.011**
Tau rhinal	-0.01 (0.05)	0.91
Tau global	0.03 (0.05)	0.52
Tau temporal	0.02 (0.05)	0.65

Standardized βs presented. All models adjusted for age, age squared, sex, time from plasma to PET, and camera. Aβ, amyloid-β. Significant associations ($p<0.05$) highlighted in bold.

Supplementary Table 3. Effect modification by amyloid status in the association between plasma tau with amyloid and tau-PET

PET measure (dependent variable)	Plasma tau (independent variable)
Amyloid FLR	0.94
Amyloid precuneus	0.80
Tau rhinal	**0.08**
Tau global	0.22
Tau temporal	0.13

p-value for interaction between plasma tau and high amyloid status. High amyloid was defined as the top decile of PiB-PET DVR. A different sample size is included in models looking at amyloid PET versus models looking at tau PET, since having both an amyloid and a tau PET scan is required in this case. In the models looking at amyloid PET outcomes, the final sample included n=375 participants with low amyloid levels and n=41 with high values. In the models looking at tau PET outcomes, the sample included n=283 participants with low amyloid levels and n=26 with high values. All models adjusted for age, age squared, sex, time from plasma to PET, camera, and their interaction with amyloid status. Associations with $p<0.1$ highlighted in bold.

Supplementary Table 4. Associations between plasma tau with tau-PET stratified by amyloid groups.

PET measure (dependent variable)	Aβ- (n=283)		Aβ+ (n=26)	
	β (SE)	p	β (SE)	p
Tau rhinal	-0.04 (0.05)	0.48	0.38 (0.34)	0.28

Standardized βs presented. High amyloid was defined as the top decile of PiB-PET DVR. All models adjusted for age, age squared, sex, time from plasma to PET, and camera. Aβ, amyloid-β. Associations with $p<0.05$ highlighted in bold.

Supplementary Table 5. Effect modification by *APOE4* status (*APOE4*+ [n=95]) versus *APOE4*- [n=317]) in the association between plasma tau with PET.

PET measure (dependent variable)	Plasma tau (independent variable)
Amyloid FLR	0.85
Amyloid precuneus	0.92
Tau rhinal	**0.03**
Tau global	0.3
Tau temporal	0.21

p-value from interaction between plasma tau and *APOE4* is presented. Significant interactions highlighted in bold. All models adjusted for age, age squared, sex, time from plasma to PET, camera used and their interactions with *APOE4*. Associations with $p<0.1$ highlighted in bold.

Supplementary Table 6. Associations between plasma tau with PET stratified by *APOE4* status.

PET measure (dependent variable)	*APOE4*- (n=234)		*APOE4*+ (n=73)	
	β (SE)	p	β (SE)	p
Tau rhinal	-0.08 (0.06)	0.21	0.20 (0.14)	0.15

Standardized βs presented. All models adjusted for age, age squared, sex, time from plasma to PET, and camera. Aβ, amyloid-β. Associations with $p<0.05$ highlighted in bold.

Supplementary Table 7. Effect modification by age group (≥57 years [n=222] versus <57 years [n=203]) in the association between plasma tau with PET

PET measure (dependent variable)	Plasma tau (independent variable)
Amyloid FLR	0.22
Amyloid precuneus	0.17
Tau rhinal	0.41
Tau global	0.51
Tau temporal	0.42

p-value from interaction between plasma tau and age group (stratified by median age of 57 years) is presented. All models adjusted for age, age squared, sex, time from plasma to PET, camera, and their interactions with age group. Aβ, amyloid-β. Associations with $p<0.1$ highlighted in bold.
